# Efficient sparse estimation on interval-censored data with approximated L0 norm: Application to child mortality

**DOI:** 10.1371/journal.pone.0249359

**Published:** 2021-04-09

**Authors:** Yan Chen, Yulu Zhao

**Affiliations:** 1 Business School, Hunan University, Changsha, Hunan, China; 2 School of Statistics and Management, Shanghai University of Finance and Economics, Shanghai, China; Yunnan University of Finance and Economics, CHINA

## Abstract

A novel penalty for the proportional hazards model under the interval-censored failure time data structure is discussed, with which the subject of variable selection is rarely studied. The penalty comes from an idea to approximate some information criterion, e.g., the BIC or AIC, and the core process is to smooth the ℓ_0_ norm. Compared with usual regularization methods, the proposed approach is free of heavily time-consuming hyperparameter tuning. The efficiency is further improved by fitting the model and selecting variables in one step. To achieve this, sieve likelihood is introduced, which simultaneously estimates the coefficients and baseline cumulative hazards function. Furthermore, it is shown that the three desired properties for penalties, i.e., continuity, sparsity, and unbiasedness, are all guaranteed. Numerical results show that the proposed sparse estimation method is of great accuracy and efficiency. Finally, the method is used on data of Nigerian children and the key factors that have effects on child mortality are found.

## Introduction

Interval-censored failure time data, which means the failure time of interest is only known to belong to a period instead of observed directly, is commonly seen in many fields, such as demography, medicine, and ecology. The statistical analysis of this special data structure has attracted much attention since first being addressed by Finkelstein (1986 [1]), and many researchers have developed significant works related to model estimations (Huang 1996 [2]; Zhang et al. 2005 [3], Zeng, Cai, and Shen 2006 [4], Lin and Wang 2010 [5]). Sun (2006 [6]) made a thorough review of research on interval-censored failure time data. Compared with right-censored data, interval-censored data can be more challenging when modeled in two ways. First, interval-censored data can be more complicated, such that sometimes it is a mixture of interval censoring and right censoring. Right censoring can be considered a special form of interval censoring with the right bound extending to infinity. Second, when implementing the proportional hazards model (Cox 1972 [7]) on right-censored data, one can use partial likelihood and does not have to estimate the baseline hazards function simultaneously with the parameters of interest.

Several methods exist that deal with interval-censored failure time data. Tong, Chen, and Sun (2008 [8]) and Goggins and Finkelstein (2000 [9]) developed approaches for interval censoring, but under strict independent assumptions. These approaches are restricted to several models; for example, the former is only applicable to the additive hazards model (Lin and Ying 1994 [10]). In this paper, a sieve maximum likelihood method with Bernstein polynomials is proposed as a general way that can be applied to many semi-parametric survival models. More information is presented below.

Although basic theories on interval-censored data are well established, studies on variable selection under this data structure are very limited. To the best of our knowledge, penalized partial likelihood has been effectively used since it is intuitive to add a penalization term to the likelihood function. Tibshirani (1997 [11]) and Fan and Li(2002 [12]) successfully applied LASSO and SCAD penalties to the proportional hazards model right after they proposed them. Zou (2006 [13]) developed adaptive LASSO (ALASSO) and Zhang and Lu (2007 [14]) used it with partial likelihood. However, penalized partial likelihood is bounded to the analysis of right-censored data. For variable selection on interval-censored failure time data, piecewise constant functions are occasionally used (Wu and Cook 2015 [15]) to represent the baseline hazards model, incorporated with several penalties and the EM algorithm is applied to optimize the likelihood function. Wang et al. (2019 [16]) introduced a Bayesian adaptive lasso penalty for the additive hazards model with Case I interval-censored data, also known as current status data, in which the subjects are only visited once and one only knows whether it has failed at the exact observation time. Zhao (2020 [17]) developed a broken adaptive ridge (BAR) penalized procedure and, with iterations, some parameters finally shrank to zero. The simulation studies show satisfying results, whereas it is still found to be computationally costly due to the heavy optimization procedures and that there are two parameters to tune.

The object of interest in the present paper is another type of covariate selection technique, i.e., best subset selection (BSS). A typical BSS method is to list all the variable subsets, model with each one of them, and use some information criterion, such as AIC (Akaike 1974 [18]) and BIC (Schwarz 1978 [19]) to judge every subset. For a dataset with *n*_0_ uncensored samples and *p* dimensions of parameters, a criterion has the form as:

$$\begin{array}{c} \min_{\beta }\varphi_{0}k-2l\left(\beta \right) \end{array}$$,

where *k*(*k* ≤ *p*) represents the number of selected parameters and *l*(⋅) represents the log-likelihood function. *φ*_0_ is fixed as 2 or *log*(*n*_0_) when a AIC or BIC is applied and it makes the criterion free of tuning, which can be a heavily time-consuming process when a common penalty such as LASSO, SCAD, or MCP (Zhang 2010 [20]) is used. The most significant problem of this method is that in the criterion a ℓ_0_ norm is involved, the discrete nature of which makes the method a NP-hard problem. Although stepwise regressions are available to help with the optimization, BSS is still infeasible when it comes to a moderately large *p*. Su et al. successfully developed an approximated form of information criterion as a penalty term [minimum information criterion (MIC)] for general linear models (GLM, 2018 [21]) and the Cox model with right-censored data (2016 [22]), which extends the BSS method to a large variable dimension.

In this study, a form of approximated information criterion is proposed under the interval-censored data structure. The result provides several major contributions to the current literature. First, an approximated BSS method is introduced into the analysis of interval-censored data, with which the variable selection approaches are rarely studied. Second, great efficiency is achieved by conducting the estimation of both the coefficients and baseline hazards function with free-tuning covariate selection simultaneously.

The rest of this paper is organized as follows. In the first section, the notation and detailed estimation procedure of interval-censored data are given. In the next section, the approximation of BSS is presented. In the third section, the simulation results of the proposed method are shown along with several other commonly seen penalties. The application section contains a survey example and the last section concludes this research and addresses a short discussion.

## Notation, assumptions, and models

### Interval censoring

Consider a failure time study that involves *n* independent subjects and for an *i*^*th*^ subject there is a *p*-dimensional vector ***Z***_*i*_(1 ≤ *i* ≤ *n*) that may affect its failure time *T*_*i*_. According to the proportional hazards model, the cumulative hazard function Λ(*t*) is given by

$$\begin{array}{c} \Lambda \left(t|Z_{i}\right)=\Lambda_{0}\left(t\right)\exp\left(Z_{i}^{\prime }\beta \right) \end{array}$$,

where Λ_0_(*t*) denotes the baseline cumulative hazard function and ***β*** = (*β*_1_, *β*_2_, …, *β*_*p*_)′ the regression coefficients. The corresponding survival function is $S\left(t|Z_{i}\right)=P\left(T_{i}\geq t|Z_{i}\right)=\exp\left(-\Lambda \left(t|Z_{i}\right)\right)=\exp\left(\Lambda_{0}\left(t\right)\exp\left(Z_{i}^{\prime }\beta \right)\right)$. Under the interval-censored data structure, observations will be recorded as **O** = {*O*_*i*_ = {*L*_*i*_, *R*_*i*_, **Z**_*i*_}, *i* = 1, …, *n*}, with (*L*_*i*_, *R*_*i*_] denoting the interval in which the failure of the *i*^*th*^ subject belongs. Then, the likelihood function can be given as

$$\begin{array}{cc} L_{n}(\beta ,\Lambda_{0}) & =\prod_{i=1}^{n}\{S\left(L_{i}|Z_{i}\right)-S\left(R_{i}|Z_{i}\right)\}\\ & =\prod_{i=1}^{n}\{\exp[-\Lambda_{0}(L_{i})\exp\left(Z_{i}^{\prime }\beta \right)]-\exp[-\Lambda_{0}(R_{i})\exp\left(Z_{i}^{\prime }\beta \right)]\}. \end{array}$$

In practice, if one does not observe the failure of some samples during the entire experiment, these samples will be considered right-censored. For right-censored subject, *R*_*i*_ = ∞ and thus *S*(*R*_*i*_|**Z**_*i*_) = 0.

To estimate *ξ* = (***β***, Λ_0_), the traditional approach is to maximize the log-likelihood function *l*_*n*_(***β***, Λ_0_), usually by finding the zeros of the derivatives. The main difficulty is to estimate finite- and infinite-dimensional parameters at the same time. This problem is discussed in the next section.

### Regularized sieve maximum likelihood estimation

To deal with the infinite-parameter estimation problem mentioned above, a sieve method (Huang and Rossini 1997 [23] and Zhou, Hu, and Sun [24]) is developed for our study. Now, consider a parameter space

$$\begin{array}{c} \Xi =\{\xi =(\beta ,\Lambda_{0})\in B⊗F\} \end{array}$$,

where $B=\{\beta \in R^{p},|\beta |\leq M\}$ with *M* a positive constant and F representing the function set that contains all bounded, continuous, non-negative, and non-decreasing functions. One common way to model λ_0_(⋅) is using splines (Wood, Pya, and Säfken 2017 [25] and Wang et al. 2019 [26]), but here, with the given restricted shape of the baseline cumulative hazards function, use of the Bernstein basis polynomials (Wang and Ghosh 2012 [27]) is preferred. Hence, a sieve parameter space

$$\begin{array}{c} \Xi_{n}=\{\xi_{n}=\left(\beta ,\Lambda_{0n}\right)\in B⊗F_{n}\} \end{array}$$

is defined, where

$$\begin{array}{c} F_{n}=\{B_{n}\left(x\right)=\sum_{j=0}^{m}{\tilde{\omega }}_{j}\cdot b_{j}\left(x,m,u,v\right):{\tilde{\omega }}_{0}\leq {\tilde{\omega }}_{1}\leq \ldots \leq {\tilde{\omega }}_{m},\sum_{j=0}^{m}|{\tilde{\omega }}_{j}|\leq M_{n}\} \end{array}$$

on the domain of observed data, recorded as [*u*, *v*]. Here, *M*_*n*_ is a positive constant and *b*_*j*_(*x*, *u*, *v*, *m*) is defined as

$$\begin{array}{c} b_{j}(x,m,u,v)=C_{m}^{j}\times (\frac{x-u}{v-u}{)}^{j}\times (1-\frac{x-u}{v-u}{)}^{m-j},\;j=0,\ldots ,m, \end{array}$$

where *m* decides the number of terms in the Bernstein basis polynomials and $\tilde{\omega }=\left({\tilde{\omega }}_{0},{\tilde{\omega }}_{1},\ldots ,{\tilde{\omega }}_{m}\right)$ denotes the coefficient vector of the terms. Note that for the non-decreasing and non-negative requirements of Λ_0*n*_, one needs $\tilde{\omega }$ following the inequality $0\leq {\tilde{\omega }}_{0}\leq {\tilde{\omega }}_{1}\leq \ldots \leq {\tilde{\omega }}_{m}$. This constraint can be ensured by reparameterization, introducing a novel vector ***ω*** = (*ω*_0_, *ω*_1_, …, *ω*_*m*_) and let ${\tilde{\omega }}_{l}=\sum_{k=0}^{l}\exp\left(\omega_{k}\right)$.

By focusing on sieve space Ξ_*n*_, the complex estimations of both infinite- and finite-dimensional parameters are converted into a much simpler estimation problem that contains only finite-dimensional parameters (***β***, ***ω***). Thus, given the argument matrix **Z** = (***Z***_1_, ***Z***_2_, …, ***Z***_*n*_), our likelihood function has the form

$$\begin{array}{c} L_{n}(\beta ,\omega |Z)=\prod_{i=1}^{n}\{\exp[-\Lambda_{0n}(L_{i})\exp\left(Z_{i}^{\prime }\beta \right)]-\exp[-\Lambda_{0n}(R_{i})\exp\left(Z_{i}^{\prime }\beta \right)]\}. \end{array}$$ (1)

To estimate parameters and select covariates simultaneously, minimizing the sieve log-likelihood function with a penalty term is considered:

$$\begin{array}{c} l_{n,pe}\left(\beta ,\omega \right)=-2log\{L_{n}(\beta ,\omega |Z)\}+\varphi \cdot pen\left(\beta \right). \end{array}$$ (2)

It is intuitive to replace *pen*(***β***) with various developed penalties. For LASSO, let $pen\left(\beta \right)=\sum_{i=1}^{p}\left|\beta_{i}\right|$; for SCAD, let $pen(\beta )=\int_{0}^{\left|\beta_{j}\right|}\text{min}\{1,{\left(a\lambda -x\right)}_{+}/(a\lambda -\lambda )\}dx$ with *a* usually fixed at 3.7; for MCP, let $pen(\beta )=\int_{0}^{\beta_{j}}{\left(1-\frac{x}{\gamma \lambda }\right)}_{+}\text{d}x$ on [0, + ∞]. However, the aforementioned penalties all need a time-consuming tuning process for hyperparameter *φ*. The approximate information criterion is introduced as a penalty term in the following section, which frees us from tuning the parameter *φ* and greatly reduces computing time.

## Approximated information criterion

### Approximation of information criterion

A novel sparse estimation method for interval-censored data is sought from the idea of approximation. The BSS method with certain information criteria does not need the parameter-tuning process, but it is infeasible for a large *p*. In this part, a smooth approximation of information criteria is developed that can be further used in the way of regularization methods.

The core task is to approximate the ℓ_0_ norm in the information criteria. The ℓ_0_ norm can be defined by indicator functions

$$\begin{array}{c} f\left(\beta \right)=\sum_{i=1}^{p}I\left(\beta_{i}\neq 0\right). \end{array}$$

The essential job is to approximate the indicator functions, for which one introduces a function *η*(*x*) that satisfies (1) *η*(0) = 0, (2) *η*(*x*) = *η*(−*x*), (3) lim_|*x*|→∞_ *η*(*x*) = 1, and (4) *η*(⋅); the latter is a smooth function and non-decreasing on $R^{+}$. Clearly, *η*(⋅) has captured the key features of the indicator *I*(*x* ≠ 0).

**Fig 1:**
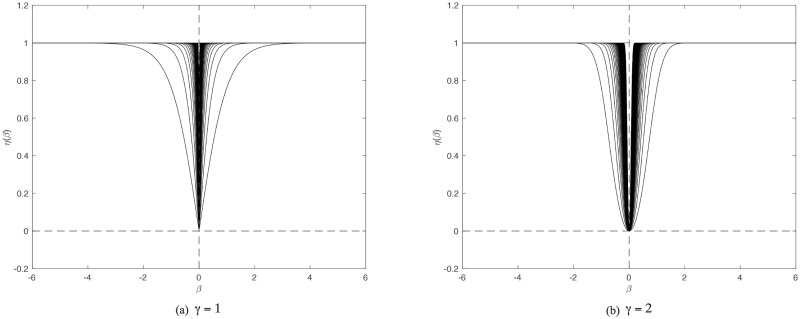
*η*(⋅) with various values of *θ*. Clearly shown is that *η*(⋅) makes a promising approximation of ℓ_0_ norm. Note that in both plots the parameter *θ* varies from 1 to 100 and the curves become sharper with larger *θ*. (a)*γ* = 1. (b)*γ* = 2.

One natural thought is to adopt sigmoid functions, which are commonly used as a smooth output unit in binary classification. The classic choice is the logistic activation sigmoid $\sigma \left(x\right)=\frac{1}{1+e^{-x}}$, and by making some minor changes on the independent variable and the intercept one can successfully develop our solution as follows:

$$\begin{array}{c} \eta \left(x\right)={2\sigma (\theta |x|}^{\gamma })-1=\frac{1-\exp(-\theta |x{|}^{\gamma })}{1+\exp(-\theta |x{|}^{\gamma })} \end{array}$$,

where *θ* > 0 and *γ* control the shape of the function. Fig 1a and 1b plot *η*(⋅) with *γ* = 1 and *γ* = 2, respectively. *θ* varies from 1 to 100. It can be seen from Fig 1b that, in general, the functions *η*(⋅) with both choices on *γ* are good approximations of *I*(*β* ≠ 0), and it seems that, with a larger *θ*, *η*(⋅) will be more like the target indicator function. Nevertheless, comparing Fig 1a and 1b, we avoid directly setting *γ* = 1 because there is a cusp at *x* = 0, although it appears to give a better performance on sparsity. This concern restricts one to set *γ* = 2. To achieve balance smoothness and sparsity together on one penalty, our motivation is to use the reparameterization procedure.

### Reparameterization

By introducing *η*(⋅), the smoothness problem of the ℓ_0_ norm is preliminarily solved by setting *γ* = 2. Fan and Li (2001 [28]) proposed three properties that a good penalty should possess: unbiasedness, sparsity, and continuity. Unbiasedness and continuity are apparently ensured by the definition. The sparsity needs to be enforced since we have chose *γ* = 2. For this purpose, the following reparameterization procedures are considered.

Set a vector $\phi ={\left(\phi_{1},\phi_{2},\ldots ,\phi_{p}\right)}^{\prime }\in R^{p}$ and relate ***ϕ*** to ***β*** by *β*_*i*_ = *ϕ*_*i*_ *η*(*ϕ*_*i*_). Define a matrix

$$\begin{array}{c} H=(\begin{array}{cccc} \eta (\phi_{1}) & 0 & \ldots & 0\\ 0 & \eta (\phi_{2}) & \ldots & 0\\ \vdots & \vdots & \ddots & \vdots \\ 0 & 0 & \ldots & \eta (\phi_{p}) \end{array}), \end{array}$$

and then the reparameterization can be written as ***β*** = ***H*** ***ϕ***. In this way, (3) is rewritten as follows:

$$\begin{array}{c} l_{n,pe}\left(\phi ,\omega \right)=-2log\{L_{n}(H\phi ,\omega |Z)\}+\varphi_{0}tr\left(H\right), \end{array}$$ (3)

where *tr*(***H***) denotes the trace of *H*. By reparameterizing *β* with *ϕ*, two goals can be achieved simultaneously: one is to keep the smoothness of the regularization problem, and the other is to obtain good performance on sparsity. These two aspects will be explained in the next section with figures.

**Fig 2:**
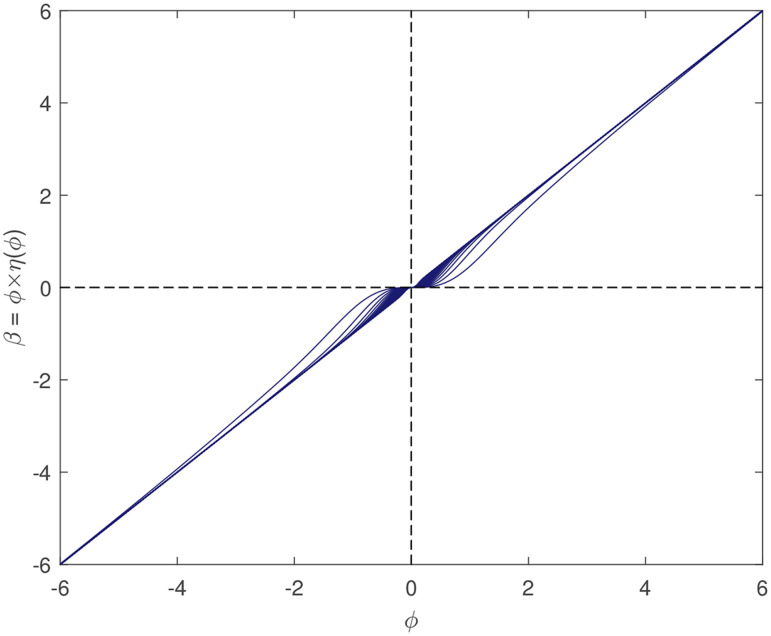
Plot of *β*_*i*_ and *ϕ*_*i*_. This plot shows desired mutually one-to-one mapping, which is strong evidence supporting reparameterizations. With *γ* varying from 1 to 100, the function becomes closer to *f*(*ϕ*) = *ϕ*.

**Fig 3:**
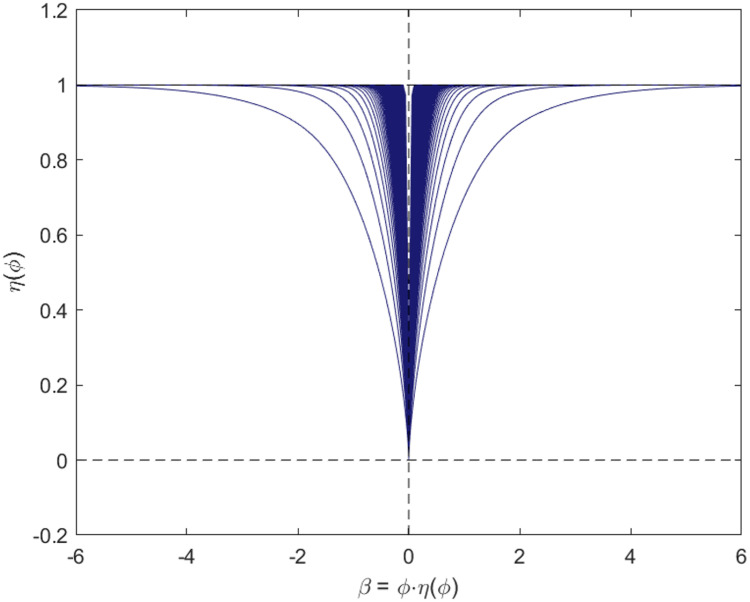
Plot of *β*_*i*_ and *η*(*ϕ*_*i*_). This plot displays relationship between true coefficients and penalty term.

1. **Smoothness.** In (4), the second term of the right-hand side *ϕ*_0_ *tr*(***H***) is smoothed by the definition of *η*(⋅) when *γ* = 2, so the problem remaining here is to check the smoothness of the first term, which is essentially decided by ***H*** ***ϕ***. ***β*** = ***H*** ***ϕ*** is composed of formula *β*_*i*_ = *ϕ*_*i*_⋅*η*(*ϕ*_*i*_), *i* = 1, …, *p*, and it is commonly known that the product of two smooth functions is also smooth. The relationship of ***β*** = ***H*** ***ϕ*** and ***ϕ*** is illustrated in Fig 2, in which a desired one-to-one mapping can be seen.
2. **Sparsity.** In the preceding section, the reason for choosing *γ* = 2 was explained, although *γ* = 1 is favorable in sparsity, which is shown in Fig 1a and 1b. Here, after reparameterization, the relationship between ***H***, which determines the penalty, and ***β***, the true coefficients, is explored. *η*(***ϕ***) and *β* are plotted in Fig 3, which demonstrates that the scale of penalty really assembles the situation when one sets *γ* = 1, in which the regularization will penalize the likelihood function with a considerably small coefficient allocated to wrong covariates.

One returns to the three properties that Fan and Li recommended, i.e., continuity, sparsity, and unbiasedness, after reparameterizing *β* with *ϕ*. Unbiasedness is ensured by the definition of *η*(⋅). Continuity lies naturally in maintaining smoothness. The goal of sparsity is attained by reparameterization, as explained in this section. Accordingly, the penalty developed herein fulfills all requirements.

Noted that after reparameterization, the penalty term becomes non-convex on ***β***. This implies the penalized log-likelihood function (4) can have multiple local optima and the initial value of optimization process will effect the result. Thus, we use simulated annealing (Belisle 1992 [29]) and BFGS quasi-Newton algorithm (see, e.g., Jorge Nocedal 1980 [30]) to obtain the final result. The simulated annealing is robust and seeks the global optimum, and BFGS algorithm is very fast and assures the result converges to a critical point. Both methods are built-in in Matlab with function simulannealbnd and fminunc. To further scrutinize our method, the numerical results are presented in the next section.

## Simulation study

An extensive simulation study is conducted in this section to assess the performance of the proposed sparse estimation method with the approximated information criterion (appIC) on finite interval-censored samples. In this study, a p-dimensional covariate set ***Z***_*p* × *n*_ = (***Z***_*i*_, ***Z***_2_, …, ***Z***_*p*_) is considered, and ***Z***_*i*_ is generated from multivariate normal distribution, with mean zero and covariance matrix **Σ**. **Σ** is defined by

$$\begin{array}{c} \Sigma =\left(\Sigma_{ij}\right),\Sigma_{ij}=0.5^{\left|i-j\right|},i,j=1,\ldots ,p \end{array}$$

Here we set three scenarios: *n* = 100, *p* = 10, *n* = 300, *p* = 10 and *n* = 300, *p* = 30. For the true coefficient vector, we set the first and last two components at *b*_0_ (*b*_0_ = 0.5 or 1), with other components zero. The baseline cumulative hazards function takes the forms Λ_0_(*t*) = *t* and Λ_0_(*t*) = *log*(*t* + 1), respectively.

When constructing interval-censored data structure, *M* visiting points are set in the interval [*u*, *v*] with a uniform gap (*v* − *u*)/*M*. To simulate the real situation in which some samples will be difficult to reach, every point is allocated a 50% chance to actually observe a certain sample. In this simulation, *u* is fixed at 0 and *v* is set as 3, thus when *b*_0_ = 0.5, there are approximately 25% and 35% right-censored portions for Λ_0_(*t*) = *t* and Λ_0_(*t*) = *log*(*t* + 1), and when *b*_0_ = 1, the portions are approximately 30% and 40%. Meanwhile, the number pf observation points *M* are set at 10 and 20, and the latter is supposed to bring more information.

**Fig 4:**
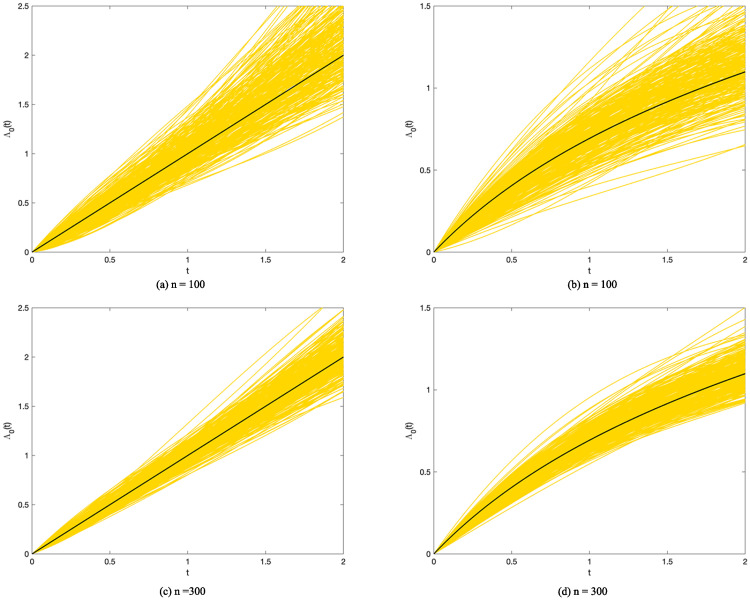
The estimated baseline cumulative functions. True and simulated baseline cumulative hazards functions shown in black and yellow, respectively. Simulated cumulative hazards function are generated with *b*_0_ = 0.5, *M* = 10 and *p* = 10. Note here (a)*n* = 100,Λ_0_(*t*) = *t*. (b)*n* = 100,Λ_0_(*t*) = *log*(*t* + 1). (c)*n* = 300,Λ_0_(*t*) = *t*. (d)*n* = 300,Λ_0_(*t*) = *log*(*t* + 1).

The hyperparameters of the proposed method are assigned as follows: (1) we set Bernstein polynomials parameter *m* = 3 because we find it sufficient to characterize the baseline cumulative hazards function; (2) we set *γ* = 2 for smoothness as described in the previous sections; (3) we fix *φ* at log(*n*_0_) (*n*_0_ denotes the number of samples that are not right-censored) as BIC; (4) we assign *θ* with *n*_0_ and the robustness of the estimate with different value of *θ* is shown in S1 File. The results are presented in Tables 1–5. In Tables 1–4, the performance is measured in three ways: mean, bias and standard deviation (SD), which indicate the accuracy and stability of our estimates. In Table 5, the performance under all scenarios are assessed with four measurements: average selected size (Size), the average true positive size (TP), the average false positive size (TP) and the median and standard deviation of ${\left(\hat{\beta }-\beta \right)}^{\prime }E\left(ZZ^{\prime }\right)\left(\hat{\beta }-\beta \right)$. It can be seen that the bias and standard deviation both greatly reduce when the sample size increases from 100 to 300. When *p* jumps to 30, appIC method remains feasible and generate good results. And the estimating accuracy and stability generally improve with a more frequent visiting pattern, which is indicated by a large *M*. Besides, it is shown that the selection correctness of the appIC method increases significantly with greater signals (*b*_0_ = 1), compared to *b*_0_ = 0.5. Meanwhile, the baseline cumulative hazards function is well modeled by Bernstein polynomials and some of the results are displayed in Fig 4. It is obvious that when *n* increases, the polynomials fit the hazards function better.

**Table 1:** Simulation result of appIC sparse estimation with *b*_0_ = 0.5 and baseline cumulative hazards function Λ_0_(*t*) = *t*.

	Mean	Bias	SD	Mean	Bias	SD
	*M* = 10			*M* = 20		
*n* = 100, *p* = 10						
*β*_1_ = 0.5	0.537	0.037	0.243	0.537	0.037	0.233
*β*_2_ = 0.5	0.494	-0.006	0.270	0.486	-0.014	0.249
*β*_9_ = 0.5	0.519	0.019	0.267	0.509	0.009	0.250
*β*_10_ = 0.5	0.501	0.001	0.251	0.509	0.009	0.215
*n* = 300, *p* = 10						
*β*_1_ = 0.5	0.510	0.010	0.092	0.513	0.013	0.084
*β*_2_ = 0.5	0.509	0.009	0.098	0.509	0.009	0.095
*β*_9_ = 0.5	0.499	-0.001	0.120	0.500	0.000	0.100
*β*_10_ = 0.5	0.510	0.010	0.100	0.507	0.007	0.088
*n* = 300, *p* = 30						
*β*_1_ = 0.5	0.524	0.024	0.101	0.518	0.018	0.093
*β*_2_ = 0.5	0.509	0.009	0.112	0.502	0.002	0.115
*β*_9_ = 0.5	0.506	0.006	0.121	0.506	0.006	0.100
*β*_10_ = 0.5	0.512	0.012	0.105	0.507	0.007	0.096

**Table 2:** Simulation result of appIC sparse estimation with *b*_0_ = 0.5 and baseline cumulative hazards function Λ_0_(*t*) = *log*(*t* + 1).

	Mean	Bias	SD	Mean	Bias	SD
	*M* = 10			*M* = 20		
*n* = 100, *p* = 10						
*β*_1_ = 0.5	0.544	0.044	0.268	0.559	0.059	0.227
*β*_2_ = 0.5	0.577	0.077	0.258	0.480	-0.020	0.265
*β*_9_ = 0.5	0.583	0.083	0.244	0.509	0.009	0.258
*β*_10_ = 0.5	0.559	0.059	0.230	0.526	0.026	0.221
*n* = 300, *p* = 10						
*β*_1_ = 0.5	0.518	0.018	0.093	0.519	0.019	0.091
*β*_2_ = 0.5	0.513	0.013	0.111	0.515	0.015	0.108
*β*_9_ = 0.5	0.504	0.004	0.119	0.506	0.006	0.109
*β*_10_ = 0.5	0.520	0.020	0.102	0.514	0.014	0.095
*n* = 300, *p* = 30						
*β*_1_ = 0.5	0.528	0.028	0.104	0.530	0.030	0.094
*β*_2_ = 0.5	0.514	0.014	0.108	0.510	0.010	0.108
*β*_9_ = 0.5	0.518	0.018	0.115	0.518	0.018	0.108
*β*_10_ = 0.5	0.517	0.017	0.110	0.515	0.015	0.104

**Table 3:** Simulation result of appIC sparse estimation with *b*_0_ = 1 and baseline cumulative hazards function Λ_0_(*t*) = *t*.

	Mean	Bias	SD	Mean	Bias	SD
	*M* = 10			*M* = 20		
*n* = 100, *p* = 10						
*β*_1_ = 1	1.152	0.152	0.276	1.132	0.132	0.251
*β*_2_ = 1	1.084	0.084	0.349	1.086	0.086	0.307
*β*_9_ = 1	1.107	0.107	0.343	1.110	0.110	0.276
*β*_10_ = 1	1.118	0.118	0.282	1.105	0.105	0.253
*n* = 300, *p* = 10						
*β*_1_ = 1	1.039	0.039	0.129	1.036	0.036	0.119
*β*_2_ = 1	1.027	0.027	0.140	1.021	0.021	0.124
*β*_9_ = 1	1.021	0.021	0.134	1.020	0.020	0.115
*β*_10_ = 1	1.035	0.035	0.136	1.028	0.028	0.122
*n* = 300, *p* = 30						
*β*_1_ = 1	1.065	0.065	0.138	1.048	0.048	0.119
*β*_2_ = 1	1.046	0.046	0.143	1.045	0.045	0.125
*β*_9_ = 1	1.046	0.046	0.133	1.041	0.041	0.121
*β*_10_ = 1	1.050	0.050	0.145	1.037	0.037	0.127

**Table 4:** Simulation result of appIC sparse estimation with *b*_0_ = 1 and baseline cumulative hazards function Λ_0_(*t*) = *log*(*t* + 1).

	Mean	Bias	SD	Mean	Bias	SD
	*M* = 10			*M* = 20		
*n* = 100, *p* = 10						
*β*_1_ = 1	1.165	0.165	0.315	1.138	0.138	0.277
*β*_2_ = 1	1.115	0.115	0.360	1.112	0.112	0.303
*β*_9_ = 1	1.155	0.155	0.355	1.143	0.143	0.314
*β*_10_ = 1	1.115	0.115	0.322	1.106	0.106	0.289
*n* = 300, *p* = 10						
*β*_1_ = 1	1.051	0.051	0.133	1.048	0.048	0.121
*β*_2_ = 1	1.043	0.043	0.148	1.036	0.036	0.128
*β*_9_ = 1	1.033	0.033	0.138	1.038	0.038	0.132
*β*_10_ = 1	1.048	0.048	0.146	1.040	0.040	0.131
*n* = 300, *p* = 30						
*β*_1_ = 1	1.068	0.068	0.141	1.071	0.071	0.132
*β*_2_ = 1	1.056	0.056	0.158	1.060	0.060	0.139
*β*_9_ = 1	1.060	0.060	0.158	1.057	0.057	0.135
*β*_10_ = 1	1.059	0.059	0.160	1.058	0.058	0.141

**Table 5:** Comparison of different scenarios with the appIC model.

	Size	TP	FP	MMSE(SD)	Size	TP	FP	MMSE(SD)
	Λ_0_(*t*) = *t*				Λ_0_(*t*) = *ln*(*t* + 1)			
*b*_0_ = 0.5								
*M* = 10								
*n* = 100, *p* = 10	3.843	3.480	0.363	0.240(0.188)	3.933	3.537	0.397	0.256(0.186)
*n* = 300, *p* = 10	4.177	3.967	0.210	0.040(0.043)	4.263	3.980	0.283	0.047(0.047)
*n* = 300, *p* = 30	4.617	3.957	0.660	0.061(0.061)	4.833	3.977	0.857	0.071(0.061)
*M* = 20								
*n* = 100, *p* = 10	3.867	3.567	0.300	0.198(0.162)	3.950	3.570	0.380	0.218(0.184)
*n* = 300, *p* = 10	4.233	3.983	0.250	0.035(0.034)	4.210	3.983	0.227	0.042(0.042)
*n* = 300, *p* = 30	4.460	3.957	0.503	0.047(0.047)	4.717	3.977	0.740	0.062(0.064)
*b*_0_ = 1								
*M* = 10								
*n* = 100, *p* = 10	4.357	3.940	0.417	0.449(0.492)	4.373	3.960	0.413	0.535(0.764)
*n* = 300, *p* = 10	4.267	4.000	0.267	0.077(0.063)	4.353	4.000	0.353	0.087(0.068)
*n* = 300, *p* = 30	4.943	4.000	0.943	0.089(0.081)	5.103	4.000	1.103	0.184(0.203)
*M* = 20								
*n* = 100, *p* = 10	4.283	3.967	0.317	0.441(0.586)	4.343	3.977	0.367	0.540(0.915)
*n* = 300, *p* = 10	4.210	4.000	0.210	0.071(0.073)	4.307	4.000	0.307	0.090(0.080)
*n* = 300, *p* = 30	4.653	4.000	0.653	0.100(0.095)	4.933	4.000	0.933	0.145(0.134)

To comprehensively assess the performance of appIC, its estimation results were compared with other commonly used approaches to variable selection: LASSO, SCAD, MCP, and BAR under the most serious conditions above, that is *M* = 10 and *b*_0_ = 0.5. The parameters of these penalties are tuned with 5-fold cross validation with the largest log-likelihood value on the validation sets. The estimations with true covariates (oracle) and full models (without selecting the covariates) are presented alongside in Tables 6 and 7. The measurements are described in the previous paragraph. It is obvious that the appIC sparse estimation performs well in both selection correctness and estimation accuracy. The variable size that it has chosen is especially close to the true size, 4, with relatively low FP. That is to say, the proposed method is very unlikely to include an irrelevant variable. Meanwhile, the TP of the appIC method rises to a satisfactory level when *n* increases from 100 to 300, performing close to SCAD and MCP. Besides, the square error of the proposed method is low with both *n* = 100 and *n* = 300. Furthermore, it is worth mentioning that our method is far faster than common penalties owing to the free-tuning on the hyperparameter. The CPU time of different methods under various conditions can be found in S1 File.

**Table 6:** Estimation results with different methods.

	Size	TP	FP	MMSE(SD)
	Λ_0_(*t*) = *t*			
*n* = 100, *p* = 10				
Full	10.000	4.000	6.000	0.694(0.814)
Oracle	4.000	4.000	0.000	0.092(0.112)
BAR	4.183	3.813	0.370	0.140(0.152)
Lasso	5.346	3.963	1.383	0.199(0.116)
Alasso	4.384	3.707	0.677	0.211(0.172)
SCAD	4.370	3.780	0.590	0.354(0.168)
MCP	4.200	3.613	0.587	0.208(0.196)
appIC	3.843	3.480	0.363	0.240(0.188)
*n* = 300, *p* = 10				
Full	10.000	4.000	6.000	0.095(0.096)
Oracle	4.000	4.000	0.000	0.028(0.030)
BAR	4.176	4.000	0.176	0.035(0.029)
Lasso	5.330	4.000	1.330	0.081(0.043)
Alasso	4.406	3.993	0.413	0.061(0.062)
SCAD	4.460	3.983	0.477	0.063(0.051)
MCP	4.393	3.993	0.400	0.031(0.041)
appIC	4.177	3.967	0.210	0.040(0.043)
*n* = 300, *p* = 30				
Full	10.000	4.000	6.000	0.256(0.211)
Oracle	4.000	4.000	0.000	0.029(0.031)
BAR	4.317	4.000	0.317	0.041(0.032)
Lasso	5.783	4.000	1.783	0.187(0.071)
Alasso	4.756	3.943	0.813	0.141(0.125)
SCAD	4.526	3.983	0.543	0.191(0.081)
MCP	4.540	3.963	0.577	0.033(0.056)
appIC	4.617	3.957	0.660	0.061(0.061)

Note that Λ_0_(*t*) = *t*.

**Table 7:** Estimation results with different methods.

	Size	TP	FP	MMSE(SD)
	Λ_0_(*t*) = *log*(*t* + 1)			
*n* = 100, *p* = 10				
Full	10.000	6.000	4.000	0.854(1.140)
Oracle	4.000	4.000	0.000	0.090(0.149)
BAR	4.236	3.753	0.483	0.181(0.192)
Lasso	5.476	3.943	1.533	0.204(0.115)
Alasso	4.750	3.727	1.023	0.218(0.151)
SCAD	4.416	3.666	0.750	0.366(0.168)
MCP	4.146	3.523	0.623	0.252(0.290)
appIC	3.933	3.537	0.397	0.256(0.186)
*n* = 300, *p* = 10				
Full	10.000	6.000	4.000	0.104(0.083)
Oracle	4.000	4.000	0.000	0.033(0.037)
BAR	4.203	4.000	0.203	0.033(0.036)
Lasso	5.253	4.000	1.253	0.070(0.045)
Alasso	4.464	3.997	0.467	0.062(0.063)
SCAD	4.400	3.997	0.403	0.081(0.056)
MCP	4.280	3.993	0.287	0.038(0.285)
appIC	4.263	3.980	0.283	0.047(0.047)
*n* = 300, *p* = 30				
Full	10.000	6.000	4.000	0.304(0.322)
Oracle	4.000	4.000	0.000	0.028(0.038)
BAR	4.191	3.994	0.197	0.038(0.039)
Lasso	6.333	4.000	2.333	0.131(0.056)
Alasso	4.633	3.920	0.713	0.172(0.162)
SCAD	4.550	3.987	0.563	0.166(0.085)
MCP	4.610	3.993	0.617	0.040(0.057)
appIC	4.833	3.977	0.857	0.071(0.061)

Note that Λ_0_(*t*) = *log*(*t* + 1).

## Application

In this section, the focus is on Nigerian child mortality data from the Demographic and Health Surveys (DHS) Program (https://www.dhsprogram.com). The dataset records women’s information from various aspects, including their children. The survey was very detailed; nevertheless, due to some practical restrictions the survival time of the children are only recorded in months or years, which makes it an interval-censored data structure. Meanwhile, it is found that the sample child mortality rate is over 20%, significantly higher than the global average, and our goal is to identify the key factors that affects children’s survival status.

A total of 24 potential factors are listed in Table 8. After excluding the samples that hold null value in either one of the variables, 8, 671 valid child samples are found, out of which 6, 830 are right censored. Note that variables 1-14 in Table 8 are dummy variables and are assigned 1 when the corresponding statements are true. Variables 15-24 are standardized so that the significance of all the factors can be compared. Most variables are concerned with the mother, and four variables marked with asterisks in Table 8 are child specific.

**Table 8:** Variables that possibly affect child mortality with six chosen by proposed sparse estimation method.

Variable number	Factor	Coefficient
V1	De facto place of residence-city	-
V2	De facto place of residence-countryside	-
V3	Has electricity	-0.110
V4	Has telephone	-1.011
V5	Presence of soap/ash/other cleansing agent in household	-
V6	Knowledge of ovulatory cycle	-
V7	Ever use of any modern contraception methods	-0.394
V8	Visited health facilities last 12m	-
V9	Smokes nothing	-
V10	Sex of household head	-
V11	When child is seriously ill, probably can not decide whether to get medical aid	0.201
V12	Child is twin*	1.185
V13	Sex of child*	-
V14	Proper body mass index	-
V15	Education in single years	-
V16	Number of household members	-0.107
V17	Number of children 5 and under	-
V18	Number of eligible women in HH	-
V19	Total children ever born	0.241
V20	Age of respondent at 1st birth	-
V21	Age at first marriage	-
V22	Ideal number of children (grp)	-
V23	Preceding birth interval*	-0.440
V24	Mother age of birth*	-

Note that four variables marked with asterisks are child specific and variables 1-14 are dummy variables, assigned as 1 when corresponding statements are true.

**Fig 5:**
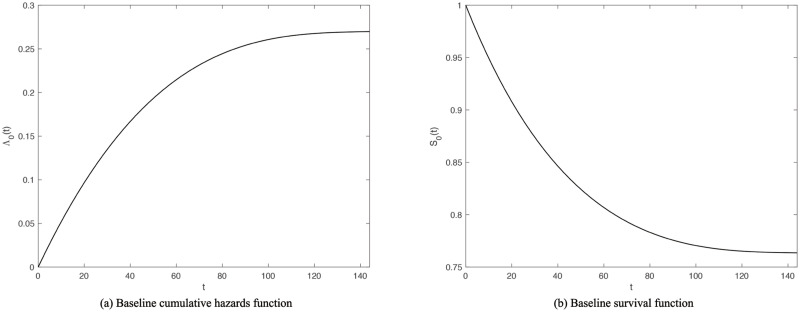
Estimated baseline cumulative hazards function and baseline survival function of children in survey. Note that *t* is measured in months. We see that the hazards function curve and survival function curve become flat as children grow up, which is consistent with reality.

To apply the appIC regression procedure here, *m* = 3, *γ* = 2, *θ* = *n*_0_ = 1841, and *φ* = log(*n*_0_) (BIC) are set. Meanwhile, the observation interval on the children is set as [0, 144]; that is to say, if the child lives to 144 months, or 12 years old, he or she is recorded as right censored. The results are shown in Table 8. According to the present research, eight variables have effects on child mortality. Having telephone, longer preceding birth interval of the mother, the usage of modern contraceptive methods, having electricity and more household members can reduce child mortality hazards, in the order of effectiveness. Meanwhile, the hazards increase with the mother having had more children and the child being twin. If the family can not decide whether to get medical aid when the child is seriously ill, the mortality hazards also increase. The baseline cumulative hazards function and baseline survival function are presented in Fig 5.

## Conclusion

In this paper, an approximated information criterion for the proportional hazards model under the interval-censored failure time data structure is discussed. The common penalties usually need a time-consuming hyperparameter tuning process, which is not necessary if one uses BSS with some well-known information criteria, such as BIC or AIC. The modified logistic sigmoid function is used herein to emulate the ℓ_0_ norm and accordingly convert the BIC as a penalized likelihood function that can be implemented in the way of regularizations. This method literally builds a bridge between BSS and the regularizations, with a special and novel strength in efficiency since it simulates the baseline hazards function, estimates coefficients of covariates, and chooses variables simultaneously, without tuning hyperparameters for the penalty term. The numerical results, including an application to child mortality, show that this method possesses great potential to facilitate mainstream sparse estimation for interval-censored data, with which the subject of variable selection is rarely studied.

There exist some interesting directions of planned future work. First, in this paper only the situation that the censoring time is independent of the failure time is considered, which sometimes may not conform with practice. Many studies discussing informative censoring exist and one can explore the proposed methods under that circumstance. The second direction is to change the survival model. Herein, only the proportional hazards model is applied, but several other superb semi-parameter survival models exist, e.g., the additive hazards model. One can compare the estimation accuracy or efficiency and show the reason. Third, in practice, some datasets with a very large covariate dimension are seen, a typical one of which is genetic data. Study on this problem is absolutely meaningful and clearly more research is needed in this direction.

## Supporting information

S1 Data (PDF)

S1 File (PDF)
