# Supplementary material for: Efficient sparse estimation on interval-censored data with approximated L0 norm: Application to child mortality
Source: PLoS One. 2021 Apr 9;16(4):e0249359. doi: 10.1371/journal.pone.0249359 (PMC8034720; doi:10.1371/journal.pone.0249359)
Supplement: S1 Data — (PDF) [file pone.0249359.s001.pdf]

Data Source

Here we describe the Demographic and Health Surveys (DHS) Program mentioned in the application section of this paper. The Demographic and Health Surveys (DHS) Program has collected, analyzed, and disseminated accurate and representative data on population, health, HIV, and nutrition through more than 400 surveys in over 90 countries. All the data in the application section is from this program.

Note that all the interested users are required to register for a download account (https://dhsprogram.com/data/available-datasets.cfm) and access the data from the approved accounts. Other users should be able to access the data set in the same manner as the authors and no contact information others would need to apply to the data.

One can download the application data in this paper by following steps:

1. Select country "Nigeria" on the webpage stated above;
2. Select the survey "Nigeria 2003";
3. Click "Survey dataset";
4. Choose "Individual Recodes" and click "Process Selected Files for Download", the ZIP file of the data set will be downloaded automatically.

The data set includes various information of the respondent women and their children. All the covariates this paper used can be found in the data set. Noted that children with missing information in those covariates are not included in the experiment, and variable 1-14 in the paper have been converted to dummy variables, which is clearly stated in the paper.
