# Supplementary material for: Efficient sparse estimation on interval-censored data with approximated L0 norm: Application to child mortality
Source: PLoS One. 2021 Apr 9;16(4):e0249359. doi: 10.1371/journal.pone.0249359 (PMC8034720; doi:10.1371/journal.pone.0249359)
Supplement: S1 File — (PDF) [file pone.0249359.s002.pdf]

1 Computation time

Table 1: **Comparison of the computation time of six models.** The experiment is implemented with $b_0 = 1$, $M = 10$ and $\Lambda_0(t) = t$, and the results (in seconds) are averaged from three simulations.

	$n = 100$		$n = 300$		$n = 1000$	
	$p = 10$	$p = 30$	$p = 10$	$p = 30$	$p = 10$	$p = 30$
CPU time						
Full	0.044	0.091	0.101	0.197	0.326	0.574
Oracle	0.027	0.037	0.054	0.074	0.091	0.136
Lasso	9.345	12.926	16.144	22.417	90.085	140.674
SCAD	40.562	48.751	110.237	134.258	-	-
BAR	73.000	109.672	244.105	446.935	-	-
appIC	1.103	1.273	1.597	3.417	3.435	4.740
Elapsed time						
Full	0.049	0.102	0.071	0.167	0.136	0.260
Oracle	0.030	0.042	0.045	0.068	0.071	0.105
Lasso	13.109	22.587	30.294	51.283	66.249	91.382
SCAD	119.578	138.562	219.695	262.781	-	-
BAR	208.039	491.932	361.207	765.268	-	-
appIC	0.715	0.737	0.776	1.190	1.735	2.478

2 Estimate $\hat{\boldsymbol{\beta}}$ with varying θ

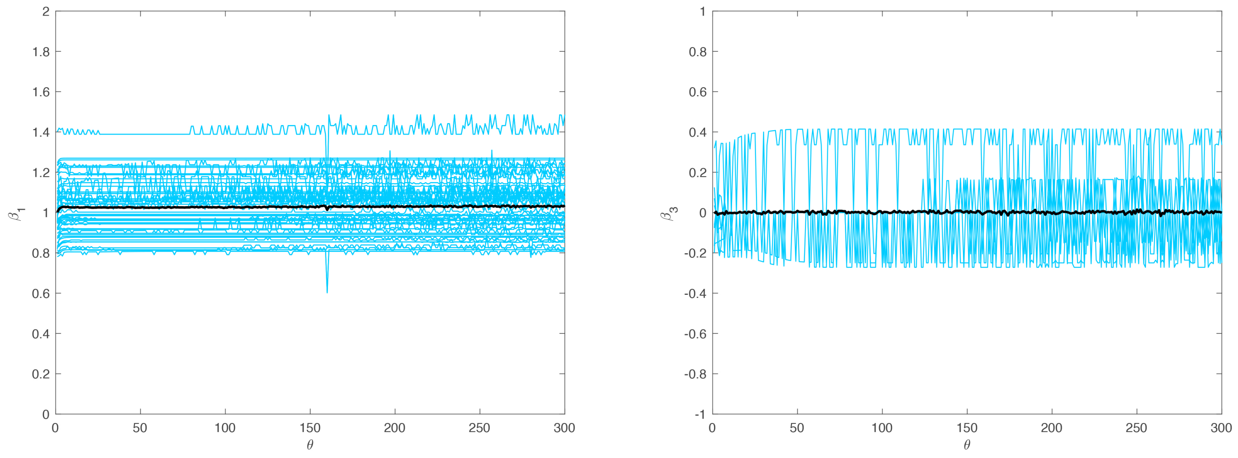

(a) (b)

Figure 1: **The value of $\hat{\beta}_1$ and $\hat{\beta}_3$ with θ varying from 1 to 300 in 50 simulations.** Note here $b_0 = 1$, $M = 10$, $n = 100$, $p = 10$ and $\Lambda_0(t) = t$, and the black line represents the average value of all simulations. We can see the estimate is quite robust with θ.
